# Assessment of the humoral immune status of varicella-zoster virus in patients with diffuse connective tissue diseases

**DOI:** 10.3389/fmed.2024.1470068

**Published:** 2024-09-05

**Authors:** Xiang Sun, Yin-shan Zang, Yan Xu, Wen Wang

**Affiliations:** ^1^Department of Expanded Program on Immunization, Jiangsu Provincial Center for Disease Control and Prevention, Nanjing, China; ^2^Department of Rheumatology and Immunology, The Affiliated Suqian First People’s Hospital of Nanjing Medical University, Suqian, China

**Keywords:** rheumatoid arthritis, diffuse connective tissue diseases, varicella-zoster virus, humoral immunity, herpes zoster, immunosuppressive therapy

## Abstract

**Background:**

Diffuse connective tissue diseases (DCTDs) require long-term immunosuppressive treatment, increasing the risk of varicella-zoster virus (VZV) infection. This study aims to evaluate the humoral immune status against VZV in DCTD patients and explore factors that may influence their immune levels.

**Methods:**

This is a retrospective cohort study that collected data from adult DCTD patients (≥18 years) attending our outpatient clinic. The geometric mean concentration (GMC) of VZV-specific IgG antibodies in the patients’ sera was measured using the enzyme-linked immunosorbent assay (ELISA).

**Results:**

A total of 280 RA patients, 272 SLE + MCTD patients and 280 healthy controls were included. SLE + MCTD patients had significantly higher VZV IgG antibody levels than RA patients (*p* < 0.05) but showed no significant difference compared to healthy controls (*p* > 0.05). Notable differences were observed particularly among female patients and those aged 30–49 years, (*p* < 0.05). SLE + MCTD patients in an active disease state had significantly higher VZV IgG antibody titers than RA patients (*p* < 0.05). Additionally, patients with a history of herpes zoster, regardless of being in the SLE + MCTD, RA, or control group, exhibited higher VZV IgG titers (*p* < 0.05).

**Conclusion:**

Although DCTD patients, particularly those with SLE and MCTD, exhibit higher VZV IgG antibody levels, they still face a higher risk of developing herpes zoster (HZ), which may be related to their underlying disease and immunosuppressive treatment. The presence of antibodies alone may not provide complete protection, necessitating consideration of cellular immune mechanisms. It is recommended to enhance monitoring of VZV antibody levels in high-risk patients and consider herpes zoster vaccination to reduce HZ-related complications.

## Introduction

Diffuse connective tissue diseases (DCTDs), including rheumatoid arthritis (RA), (1), systemic lupus erythematosus (SLE) (2) and mixed connective tissue disease (MCTD) (3), are chronic autoimmune conditions characterized by multisystem involvement and immune-mediated damage. These diseases often require long-term immunosuppressive therapy, increasing the risk of infections, particularly those caused by the varicella-zoster virus (VZV).

VZV, a neurotropic DNA virus (4, 5), causes chickenpox and can reactivate as herpes zoster (HZ) during periods of immunosuppression (6). DCTD patients are particularly susceptible to VZV reactivation due to their immunocompromised state, which can lead to severe complications and exacerbate their underlying condition (7–9).

Recent research has shed light on the humoral immune status of VZV in DCTD patients. Krasselt et al. (10) found that patients with SLE and RA had altered humoral immunity to VZV compared to healthy controls. Their study revealed lower VZV-specific IgG levels in RA patients, while SLE patients showed no significant difference from controls. However, the implications of these findings for HZ risk and the impact of various treatment regimens remained unclear. In particular, there is a lack of in-depth understanding of several key issues: whether there is a difference in VZV-specific IgG antibody levels between DCTD patients and healthy individuals, whether significant differences exist in VZV immune status among patients with different types of DCTDs [RA (11), SLE (10) and MCTD], how factors such as disease duration, disease activity, and immunosuppressive treatment (12–15) affect VZV immune levels in DCTD patients, and whether the VZV immune status of DCTD patients is related to their risk of developing herpes zoster. Understanding the VZV immune status of this special population is of great significance for formulating prevention strategies and optimizing treatment plans. Therefore, this study aims to evaluate the humoral immune status of DCTD patients against VZV through a retrospective cohort study and explore factors that may influence their immune levels. We will also compare the VZV antibody levels of these patients with those of a healthy control group to determine whether DCTD patients have a higher risk of herpes zoster. Through this study, we hope to provide a scientific basis for the prevention, diagnosis, and treatment of DCTD patients, thereby improving their quality of life and prognosis.

## Methods

### Study design and participants

Over an eight-month period, all adult DCTD patients (≥18 years) attending routine consultations at our outpatient clinic were invited to participate in this retrospective cohort study. There were no further restrictions on gender, age, or specific treatments to ensure a representative sample of outpatients. Diagnoses were based on the clinical judgment of rheumatologists, typically following the classification criteria established by the American College of Rheumatology (ACR) and the European League Against Rheumatism (EULAR) (16) in 2010. DCTD activity, mainly according to the last disease activity score 28, Systemic Lupus Erythematosus disease activity index and EULAR primary Sjögren’s syndrome disease activity index score, or rheumatologists criteria. The condition is classified as stable with no activity and mild activity, while other patients are classified as active. The treatment drug regimen for enrolled patients has not undergone significant adjustments in the past year. Clinical data and herpes zoster history were taken during the visit or was available from the existing medical records. Herpes zoster infection occurred within the past year. Vaccination data was sourced from the Jiangsu Provincial Integrated Service Management Information System for Vaccination. Healthy controls were randomly selected from routine hospital diagnoses, matched by age and gender to the case group.

### Serum collection and serological testing

Approximately 5 mL of venous blood was drawn from each participant and stored at −70°C until analysis. VZV-specific IgG antibodies were measured using ELISA at the central laboratory of the Affiliated Suqian First People’s Hospital of Nanjing Medical University. The quantification of varicella immunoglobulin G (IgG) antibodies’ geometric mean concentrations (GMC) is conducted through glycoprotein-based enzyme-linked immunosorbent assay (gpELISA). The ELISA kit used (from Institut Virion/Serion GmbH) measures specific IgG class antibodies against the viral envelope glycoprotein of VZV bound to microtitration wells. A positive result was defined as a varicella virus IgG antibody concentration ≥50 mIU/mL.

### Statistical analysis

Statistical analysis was performed using SPSS v22.0. Continuous data were described using means (M) and standard deviations (SD), while categorical data were described using absolute or relative frequencies. Fisher’s exact test was employed to compare categorical variable frequencies. For continuous data comparisons, normality tests were followed by student’s *t*-test or Mann–Whitney *U* test. A *p*-value <0.05 was considered statistically significant.

## Results

A total of 280 RA patients (mean age 50.0 ± 13.3 years) and 272 SLE + MCTD patients (with SLE accounting for approximately 50%, mean age 49.5 ± 14.3 years) were included. Additionally, 280 healthy controls (mean age 50.1 ± 10.2 years) were randomly selected from routine diagnoses. None of the participants had received the herpes zoster vaccine. Detailed information about the included patients and control group is presented in Table 1, and the medication details for the patient group are shown in Table 2. There were no significant differences in age and gender between the patient and healthy control groups.

**Table 1:** Characteristics of the studied patients and controls.

	SLE + MCTD (*N* = 272)	RA (*N* = 280)	Control (*N* = 280)	F/P
VZV antibody concentration (mIU/mL)	2936.1 (1982.0–3890.2)	1774.8 (1343.1–2206.5)	2960.7 (2466.2–3455.2)	0.017
Positive rate of VZV (%)	95.6	97.1	98.9	0.057
Mean age (years, M ± SD)	49.5 ± 14.3	50.0 ± 13.3	50.1 ± 10.2	0.826
Duration of disease (years, M ± SD)	5.3 ± 4.7	8.3 ± 7.6	NA	0.000
Female *n* (%)	212(77.9%)	216(77.1%)	226(81.0%)	0.501
HZ history *n* (%)	22(8.1%)	9(3.2%)	8(2.9%)	0.005
Mean time from disease onset to HZ (years, M ± SD)	3.3 ± 1.9	6.4 ± 2.8	NA	0.002

**Table 2:** Medication of the studied patients with RA and ODCTD.

Characteristics	RA (*n* = 280)	SLE + MCTD (*n* = 272)
Medication, *n* (%)		
NSAIDs	117 (41.8%)	9 (3.3%)
csDMARDs, *n* (%)		
GC	61 (21.8%)	165 (60.7%)
HCQ	123 (43.9%)	201 (73.9%)
MTX	145 (51.8%)	12 (4.4%)
SSZ	2 (0.7%)	0 (0%)
CTX	2 (0.7%)	7 (2.6%)
LEF	52 (18.6%)	15 (5.5%)
AZA	0 (0%)	8 (2.9%)
MMF	1 (0.4%)	58 (21.3%)
CsA	3 (1.1%)	13 (4.8%)
FK506	1 (0.4%)	35 (12.9%)
bDMARDs, *n* (%)		
TNFi	24 (8.6%)	0 (0%)
RTX	0 (0%)	2 (0.7%)
IL-6i	14 (5.0%)	0 (0%)
BlySi	0 (0%)	21 (7.7%)
IL-17i	0 (0%)	0 (0%)
tsDMARDs, *n* (%)		
JAKi	69 (24.6%)	8 (%)

NSAIDs, nonsteroidal antiinflammatory drugs; GC, glucocorticoid; DMARDs, disease-modifying anti-rheumatic drugs; csDMARDs, conventional synthetic disease-modifying antirheumatic drugs; HCQ, hydroxychloroquine; MTX, methotrexate; SSZ, sulfasalazine; CTX, cyclophosphamide; LEF, leflunomide; AZA, azathioprine; MMF, mycophenolate mofetil; CsA, cyclosporine; FK506, tacrolimus; bDMARDs, biologic disease-modifying antirheumatic drugs; TNFi, tumour necrosis factor inhibitors; RTX, rituximab; IL-6i, IL-6 inhibitors; BlySi, belimumab; IL-17i, IL-17 inhibitors; tsDMARDs, targeted synthetic disease-modifying antirheumatic drugs; JAKi, Janus Kinase inhibitors.

**Figure 1:**
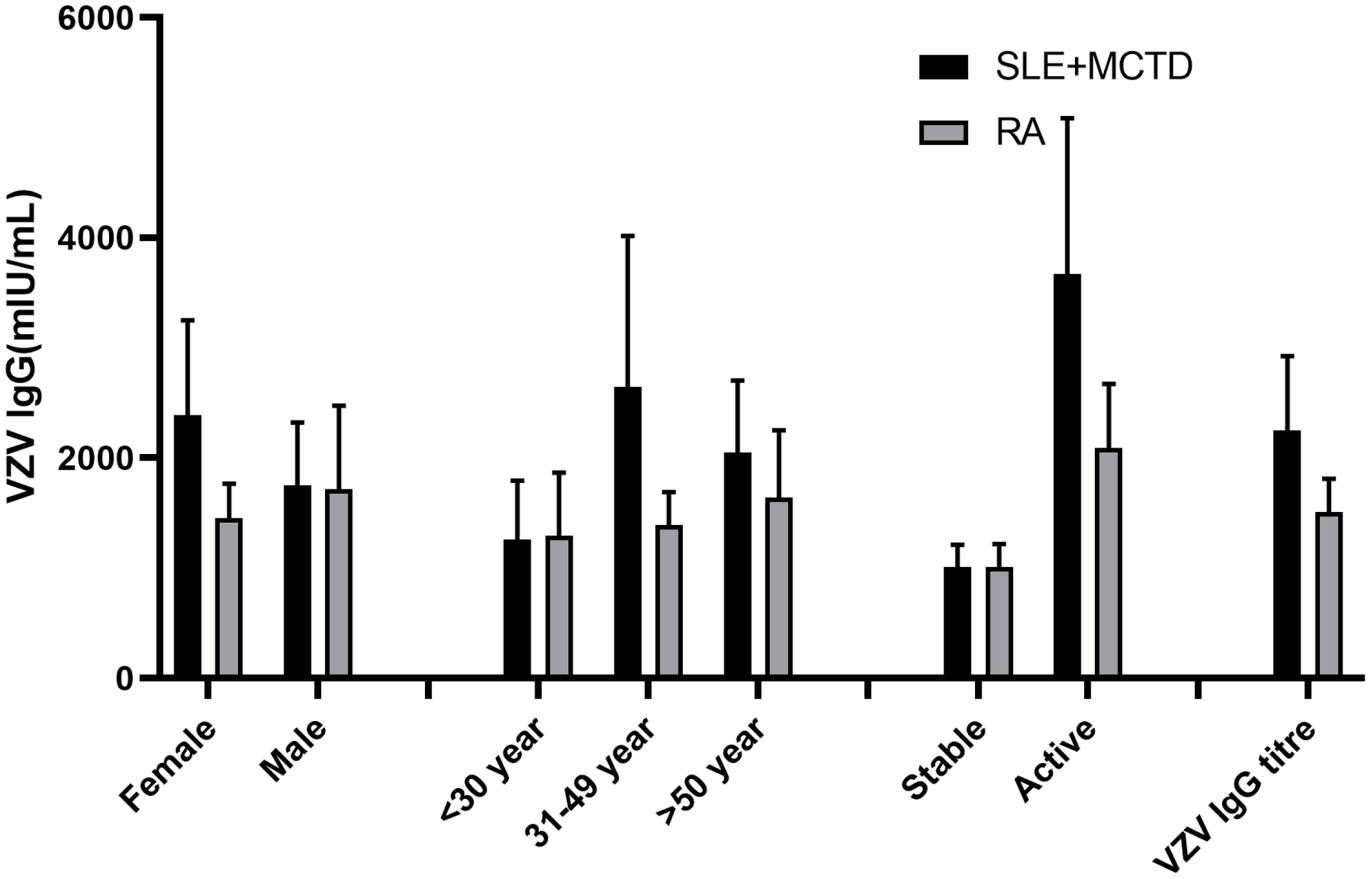
Comparison of VZV antibody titers between patients with RA and SLE + MCTD (excluding HZ).

The VZV IgG antibody titers in SLE + MCTD patients were slightly lower than those in the control group (*p* > 0.05) but significantly higher than in RA patients (*p* = 0.017). Additionally, the VZV IgG antibody positivity rate in the control group was higher than in both RA patients and SLE + MCTD patients (*p* = 0.057). Stratified analysis (excluding those with a history of herpes zoster) showed that among the two patient groups (SLE + MCTD and RA), the VZV IgG concentration in SLE + MCTD patients treated with a combination of glucocorticoids and conventional + biological DMARDs (GC/csDMARDs + bDMARDs) was significantly higher than that in RA patients (*p* < 0.05). Age stratification revealed that ODCTDs patients aged 30–49 had higher VZV IgG antibody titers compared to RA patients (*p* < 0.05), while no statistically significant differences were observed in other age groups (*p* > 0.05). Furthermore, when the disease was active, the VZV IgG antibody titers in the SLE + MCTD patient group were 3670.9 (2255.8–5086.0) mIU/mL, significantly higher than in the RA patient group, which had titers of 2092.9 (1513.1–2672.6) mIU/mL (*p* < 0.05) (Figure 1).

In our cohort, the average age at which SLE + MCTD patients developed herpes zoster (HZ) was 46.4 ± 13.3 years, compared to 42.3 ± 11.2 years for RA patients. The incidence rates of HZ in the SLE + MCTD patient group, the RA patient group, and the control group were 8.1, 3.2, and 2.9%, respectively (*p* < 0.05). The average time from the onset of rheumatic disease to the development of HZ was shorter in SLE + MCTD patients compared to RA patients (3.3 ± 1.9 vs. 6.4 ± 2.9 years, *p* < 0.05, Table 1). When comparing the VZV IgG levels between SLE + MCTD patients with positive HZ history and any other group (including SLE + MCTD patients without HZ history, but also RA and controls groups), SLE + MCTD patients show higher titers after at least one episode of HZ within 2 years (Supplementary Table S1). In the SLE + MCTD patient group, the use of glucocorticoids, regardless of dosage, was associated with a higher risk of developing HZ (*p* < 0.05).

## Discussion

This study aimed to assess the humoral immune status against VZV in patients with DCTD and identify factors that may influence the risk of VZV infections. Our findings not only confirm the significantly increased risk of HZ in DCTD patients (15, 17, 18), particularly those with SLE, MCT and RA, but also reveal how advancing age and the use of immunosuppressive agents further exacerbate this risk (15, 19). We observed significant differences in VZV IgG antibody levels between DCTD patients and healthy controls, particularly within the RA and SLE + MCTD subgroups, where variations in treatment regimens and disease activity are notably prominent. Interestingly, patients with SLE + MCTD exhibited significantly higher VZV IgG antibody levels compared to RA patients (*p* = 0.017), though not significantly different from the control group (*p* > 0.05). This aligns with the findings of Yin and Wen (20) and Wang et al. (21), who suggested that impaired immune system function, particularly abnormal B and T cell activity, may lead to reduced antibody production in RA patients.

Moreover, the immunosuppressive medications commonly prescribed to RA patients, while effective in controlling disease activity, also suppress normal immune function. Consequently, different treatment regimens may impact patients’ VZV immune status. Our study found that RA patients undergoing methotrexate treatment had lower antibody levels, potentially due to the immunomodulatory effects of these drugs, which might inhibit antibody production (not shown in results). Interestingly, although SLE + MCTD patients had higher VZV IgG levels than RA patients, they did not significantly differ from healthy controls (*p* > 0.05). This may suggest that SLE + MCTD patients, despite having relatively preserved humoral immunity, still face elevated HZ risks, likely due to their underlying disease and treatment protocols. However, subgroup analysis revealed that disease activity is a significant factor influencing VZV IgG levels. Patients with active disease exhibited statistically higher VZV IgG levels. Additionally, we found that patients with active disease were more likely to receive biologic therapy. Future research should consider prospective designs, larger sample sizes, and more detailed treatment histories to better understand the relationship between disease activity, different treatment regimens (including biologics), and VZV immunity.

Disease activity significantly influences VZV IgG levels, with DCTD patients exhibiting higher antibody titers during active disease phases. This likely reflects enhanced immune activation associated with disease flares, which promotes increased antibody production. Specifically, SLE + MCTD patients with active disease demonstrated significantly higher VZV IgG levels compared to RA patients, suggesting distinct immunodynamic responses in SLE + MCTD, possibly related to elevated inflammation during disease activity. The inflammatory milieu during active disease phases may alter immune cell function, thereby affecting the activation of VZV-specific B cells and subsequent antibody production. This finding is consistent with Nagasawa et al. (22), who observed common occurrences of polyclonal hypergammaglobulinemia in SLE patients, potentially due to B cell activation (22–24).

In our study, we found that patients with a history of HZ, regardless of their DCTD diagnosis, had significantly higher VZV IgG titers compared to those without such history. This finding aligns with the concept of endogenous boosting, where VZV reactivation stimulates antibody production. As noted by Nagasawa et al. (22), who measured antibodies using a neutralization test, at least one episode of HZ is related to higher VZV titers in SLE patients. Importantly, our results showed that SLE + MCTD patients with higher titers after at least one episode of HZ within 2 years. While these findings emphasize the impact of a prior HZ on the VZV antibody levels in SLE patients, they also imply the existence of SLE + MCTD specific factors. Polyclonal hypergammaglobulinemia, frequently seen in patients with SLE (23, 25), was initially thought to explain high VZV IgG levels in SLE + MCTD patients (22, 26). However, this assumption was questioned when IgG antibodies against diphtheria were found to be lower in SLE + MCTD patients than in age-matched healthy controls, while VZV IgG antibodies were increased in the same SLE cohort (8). Recent research suggests that the high levels of VZV antibodies in SLE + MCTD patients might be the combined consequence of both autoreactive B-cells and prior HZ (27, 28). These findings underscore the complexity of interpreting VZV serology in DCTD patients. While higher VZV IgG levels might initially seem protective, they may instead reflect a history of VZV reactivation, which paradoxically indicates a higher risk for future episodes. This highlights the importance of considering clinical history alongside serological data when assessing HZ risk in these patients.

This study has some limitations. Firstly, while our study shares similarities with previous work by Krasselt et al. (10), we offer important advancements. Our larger sample size, inclusion of MCTD patients, and more comprehensive analysis of factors like disease activity and treatment regimens provide a broader and more nuanced understanding of VZV immunity in DCTD patients. Secondly, cellular immunity plays a more critical role in protecting against HZ. In the future, increasing the measurement of cellular-mediated immunity (CMI) may be more valuable for predicting HZ. Thirdly, as a retrospective single-center study, there are potential biases related to medical records and patient recall, which may limit the generalizability of the results to broader populations. Lastly, while our sample size is larger than previous studies, further multi-center investigations with even larger cohorts could help validate and extend our findings.

In conclusion, this study underscores the importance of understanding the humoral immune status of VZV in patients with DCTD. Despite higher VZV IgG levels, SLE + MCTD patients remain at substantial risk for VZV reactivation, likely due to underlying disease mechanisms and immunosuppressive treatments. These findings suggest that antibody presence alone is insufficient for protection, highlighting the need for comprehensive immune monitoring and tailored prophylactic strategies. Clinicians should consider preventive measures, including HZ vaccination, to mitigate the risk of VZV reactivation and associated complications in these high-risk populations.

## Data Availability

The raw data supporting the conclusions of this article will be made available by the authors, without undue reservation.
