# Supplementary material for: Assessment of the humoral immune status of varicella-zoster virus in patients with diffuse connective tissue diseases
Source: Front Med (Lausanne). 2024 Sep 5;11:1470068. doi: 10.3389/fmed.2024.1470068 (PMC11410609; doi:10.3389/fmed.2024.1470068)
Supplement: Supplementary file 1 [file Data_Sheet_1.docx]

Table Comparison of VZV antibody titers between patients and controls

|  | SLE+MCTD (N=272) | RA (N=280) | Control (N=280) | P |
| --- | --- | --- | --- | --- |
| HZ history | 40.011/0.000 | 50.998/0.000 | 5.165/0.024 |  |
| No | 2085.9(1498.8-2673.1) | 1512.0(1214.2-1809.0) | 2863.5(2367.6-3359.4) | 0.000 |
| Yes | 12597.5 (3145.1-22049.9) | 9688.6(837.4-20214.7) | 6265.1(2229.9-10300.2) | 0.677 |
